# Comparison of Antioxidant Properties of a Conjugate of Taxifolin with Glyoxylic Acid and Selected Flavonoids

**DOI:** 10.3390/antiox10081262

**Published:** 2021-08-08

**Authors:** Victoria S. Shubina, Victoria I. Kozina, Yuri V. Shatalin

**Affiliations:** Institute of Theoretical and Experimental Biophysics, Russian Academy of Sciences, Institutskaya 3, 142290 Pushchino, Russia; ernie.nike@yandex.ru (V.I.K.); yury.shatalin@yandex.ru (Y.V.S.)

**Keywords:** flavonoids, glyoxylate-taxifolin condensation product, antioxidant activity, metal-reducing ability, iron-chelating activity, ferrozine

## Abstract

It is known that flavonoids can react with toxic carbonyl compounds in the process of the storage, aging, and digestion of flavonoid-rich foods and beverages. However, the effect of these reactions on the antioxidant properties of the polyphenolic fraction and the properties of the resulting products remain poorly studied. The aim of the present work was to study the antioxidant activity of quercetin, taxifolin, catechin, eriodictyol, hesperetin, naringenin and a product of the condensation of taxifolin with glyoxylic acid, as well as to reveal the structure–activity relationship of these polyphenols. It was found that flavonoids containing the catechol moiety exhibited higher antioxidant activity than hesperetin and naringenin. The product showed the highest hydrogen peroxide scavenging activity, a lower metal-reducing and a higher iron-binding ability than catechol-containing flavonoids, and a lipid peroxidation inhibitory activity comparable with that of taxifolin. Thus, the condensation of flavonoids with toxic carbonyl compounds might lead to the formation of products exhibiting high antioxidant activity. Meanwhile, the conditions under which parent flavonoids and their products exhibit the maximal antioxidant activity may differ. The data suggest that the antioxidant profile of the polyphenolic fraction and bioavailability of polyphenols, carbonyl compounds, and metal ions may change when these reactions occur.

## 1. Introduction

Polyphenols are the most numerous and widely distributed compounds of plant origin [1]. They are involved in various processes of the growth and development of plants [2], and their protection against unfavorable environmental factors [3,4,5]. They enter the body of humans and animals with plant food. The intake of polyphenols or polyphenol-rich food products might be associated with a lower risk of cardiovascular [6,7,8], neurodegenerative [9,10,11,12], and other diseases [6,13,14]. More than 8000 polyphenols have been identified; of these, more than 4000 compounds belong to the group of flavonoids [1]. In nature, polyphenols occur as monomers, oligomers, and polymers (proanthocyanidins, condensed tannins). There is also evidence indicating that, during the storage and aging of food products and beverages with a high content of flavonoids, the latter react with carbonyl compounds such as acetaldehyde, methylglyoxal, glyoxylic acid, and furfurol, which results in the formation of monomeric, oligomeric, and polymeric adducts [15,16,17,18,19,20,21,22].

When entering the human gastrointestinal tract (GIT), polyphenols either remain in the native form or undergo various chemical transformations [23]. Both parent and modified polyphenols are capable of producing local effects. In addition, when entering the circulation, they may induce systemic effects [23]. In the first case, the effect of polyphenols can be appreciable because their concentration in the GIT can reach rather high values [23,24,25]. In particular, they can cause antioxidant effects by interacting with reactive oxygen species (ROS) and ions of metals of variable valency or react with toxic compounds formed during the preparation and digestion of food. This is evidenced by the literature data indicating that the consumption of some food products containing partially oxidized lipids increases the level of toxic hydroperoxides and malonic aldehyde in the stomach and, as a result of their absorption, in the blood plasma. The combined consumption of these food products with polyphenolic compounds substantially reduces or completely prevents the accumulation of these toxic compounds [26,27,28,29,30], producing a protective effect on the body. However, the properties of the resulting compounds as well as the effect of the condensation of polyphenols with carbonyl compounds on the antioxidant properties of the polyphenolic fraction remain poorly understood.

**Figure 1:**
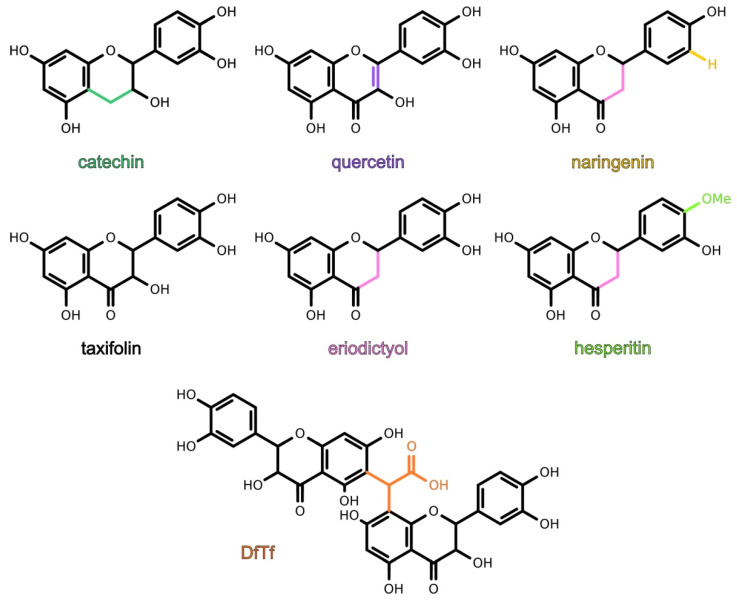
Structures of polyphenols being tested. The product of the condensation of taxifolin with glyoxylic acid is designated as DfTf.

The goal of the present work was to study the antioxidant activity of six structurally similar flavonoids (quercetin, taxifolin, catechin, eriodictyol, hesperetin, naringenin) and a product of the condensation of taxifolin with glyoxylic acid (Figure 1), as well as to reveal the structure–activity relationship of these polyphenols. For this purpose, the ability of the polyphenols to scavenge hydrogen peroxide and inhibit iron-induced lipid peroxidation was examined. In addition, the ability of the polyphenols to reduce iron and cooper ions was estimated.

## 2. Materials and Methods

### 2.1. Chemicals

Taxifolin, quercetin, catechin, eriodictyol, hesperetin, naringenin, horseradish peroxidase (150 U/mg), 3-(2-pyridyl)-5,6-diphenyl-1,2,4-triazine-4,40-disulfonic acid monosodium salt hydrate (ferrozine), bathocuproinedisulfonic acid (BCDS), copper (II) sulfate, ascorbic acid, 1-butanol, 1,1,3,3 tetraethoxypropane, and 5-amino-2,3-dihydro-1,4-phthalazinedione (luminol) were obtained from Sigma-Aldrich (St. Louis, MO, USA). Acetic acid, orthophosphoric acid (H_3_PO_4_), sodium acetate, ferrous sulfate (FeSO4·7H_2_O), ferric chloride (FeCl_3_·6H_2_O), and hydrogen peroxide (H_2_O_2_) were purchased from Reakhim (Moscow, Russia). Phosphate buffered saline (PBS) was purchased from Paneco (Moscow, Russia), thiobarbituric acid (TBA) from Dia-m (Moscow, Russia), and lecithin from AppliChem (Darmstadt, Germany). The product of the condensation of taxifolin with glyoxylic acid (DfTf) was synthesized as described previously [31]. All reagents were of analytical grade purity. Water used for the preparation of the solutions was purified by a Milli-Q system (Millipore, Burlington, MA, USA).

### 2.2. Hydrogen Peroxide Scavenging Activity

The hydrogen peroxide scavenging activity of polyphenols was estimated by luminol-dependent chemiluminescence (LCL) as described previously [31]. Briefly, the reaction mixtures contained NaH_2_PO_4_/NaOH buffer (20 mM, pH 7.4), luminol (100 µM), horseradish peroxidase (1.25 U), hydrogen peroxide (10 µM), and polyphenols at different concentrations (0.25–40.0 µM). Hydrogen peroxide was added to the solutions immediately prior to the registration of the signal. LCL was recorded in 96-well plates (Greiner, Kremsmunster, Austria) using a multi-plate reader (Infinite F200 Tecan, Grodig, Austria) until it returned to the baseline. The reduction in LCL signal intensity in the presence of polyphenols was estimated using the following formula: ∫LCL = (∫polyphenol/∫control) × 100%, where ∫LCL is the integral LCL, %; ∫polyphenol is the integral LCL in the presence of polyphenols; ∫control is the integral LCL without the addition of polyphenols. Measurements were carried out at 37 °C.

### 2.3. Metal-Reducing Activity

#### 2.3.1. Copper-Reducing Activity

The copper-reducing ability of polyphenols was assayed using BCDS [32]. Briefly, the reaction mixtures contained NaH_2_PO_4_/NaOH buffer (20 mM, pH 7.4), BCDS (600 µM), copper (II) sulfate (100 µM), and polyphenols at different concentrations (5–130 µM). CuSO_4_ was added to the mixtures immediately prior to measurements. The Cu (I)-BCDS complex was detected at 483 nm at 37 °C. Measurements were carried out on a multi-plate reader (Infinite F200 Tecan) in 96-well plates (Greiner). The concentration of Cu^+^ was evaluated by a calibration curve obtained using the solutions of CuSO_4_ and ascorbic acid (200 µM); the latter served as a standard reducing agent [32].

#### 2.3.2. Iron-Reducing Activity

The iron-reducing ability of polyphenols was evaluated using ferrozine [32]. Briefly, the reaction mixtures contained Na-acetate buffer (20 mM, pH 5.0), ferrozine (1 mM), iron (III) chloride (100 µM), and polyphenols at different concentrations (5–130 µM). FeCl_3_ was added to the mixtures immediately prior to measurements. The Fe (II)-ferrozine complex was detected at 562 nm at 37 °C. Measurements were carried out on a multi-plate reader (Infinite F200, Tecan) in 96-well plates (Greiner). The Fe^2+^ concentration was estimated by a calibration curve obtained using FeSO_4_ solutions.

### 2.4. Iron-Binding Properties of DfTf

Competition between the product and ferrozine for Fe^2+^ was assessed as follows. A solution of FeSO_4_ (at a final concentration of 0.1 mM) was first added to the solutions of DfTf dissolved in PBS (at the final concentrations of 0.16, 0.31, 0.63, 1.25, 2.50, and 5.00 mM). This led to the formation of a polyphenol-iron complex, which was detected at 460 nm [31]. After 30 min, a solution of ferrozine (at a final concentration of 333 µM) was added to the mixtures. As a strong chelator, ferrozine bound free ions and ions weakly bound by polyphenol. The resulting Fe(ferrozine)_3_^2+^ complex was recorded at 562 nm. On the basis of the data on absorption changes (at 562 nm) depending on the molar ratio DfTf/ferrozine, the apparent binding constant of the DfTf-iron complex was estimated. The calculation of the apparent binding constant is given in Supplementary Materials. Experiments were performed in 96-well microplates at 37 °C using a Tecan Infinite F200 microplate reader.

### 2.5. Lipid Peroxidation Inhibitory Activity

Lipid peroxidation was estimated by measuring the content of malondialdehyde (MDA). The production of MDA was measured using TBA [33,34]. Briefly, the reaction mixtures containing hydrogen peroxide (1 mM), FeSO_4_ (1 mM), lecithin (10 mg/mL), and polyphenols at different concentrations (0, 0.03, 0.06, 0.12, 0.24, 0.50, 1.00, 2.00, 4.00 mM) were incubated at 37 °C for 1 h. Then, 0.50 mL of TBA (0.67%) dissolved in 2% orthophosphoric acid was added to 0.50 mL of the mixtures. After that, the mixtures were incubated at 100 °C for 1 h. After cooling, the resulting colored adduct was extracted from the mixtures with 1.0 mL of *n*-butanol. At the end, the adduct was detected using a Cary 100 Scan spectrophotometer (Varian, Sydney, Australia) at 532 nm. 1,1,3,3-Tetraethoxypropane was used as an MDA standard.

### 2.6. Statistical Analysis

The data represent the means ± standard deviation of at least five independent experiments. The statistical significance was estimated by the Student’s *t*-test (*p* < 0.05).

## 3. Results

### 3.1. Hydrogen Peroxide Scavenging Activity 

**Figure 2:**
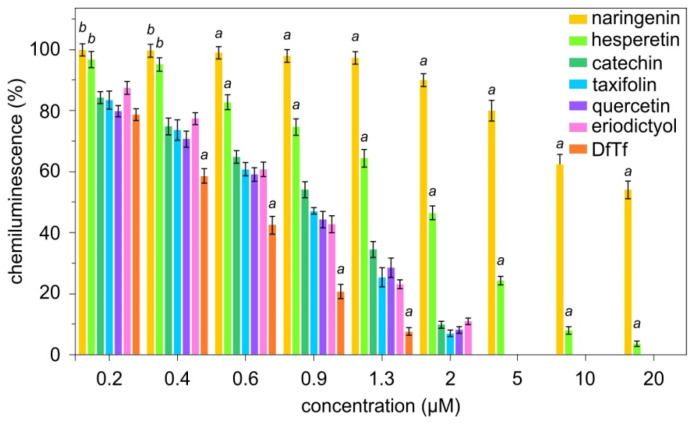
Effect of polyphenols on chemiluminescence. ^a^ differences are significant compared with other compounds; ^b^ differences are significant compared with catechol-containing compounds, *p* < 0.05.

It was found that the integral LCL response in the presence of polyphenols decreases with increasing concentration of these compounds (Figure 2). Polyphenol concentrations necessary for the 50% inhibition of LCL (IC_50_) are given in Table 1. It is seen that DfTf exhibits the highest antioxidant activity. Next are eriodictyol, quercetin, taxifolin, and catechin, which show similar antioxidant properties. It should be noted that these compounds contain two hydroxyl groups in the B ring, which are in the ortho-position relative to each other (the catechol fragment). Hesperetin and naringenin exhibit a lower antioxidant activity.

### 3.2. Metal-Reducing Activity

**Figure 3:**
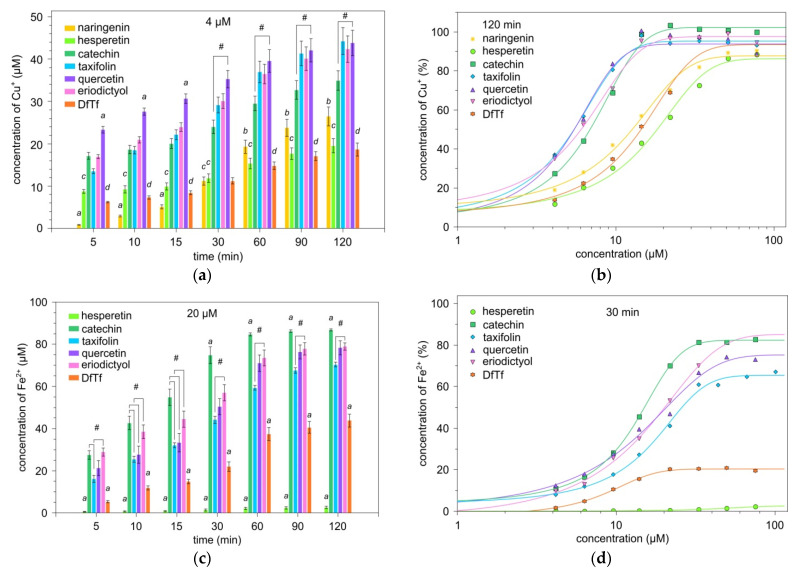
Metal-reducing activity of polyphenols. Reduction of cupric (**a**,**b**) and ferric (**c**,**d**) ions depending on the time (**a**,**c**) and concentration of polyphenols (**b**,**d**). ^a^ differences are significant compared with other compounds; ^b^ differences are significant compared with catechol-containing compounds; ^c^ differences are significant compared with catechol-containing compounds, with the exception of DfTf; ^d^ differences are significant compared with other compounds, except hesperetin; ^#^ significant differences between the compounds, *p* < 0.05.

Figure 3 shows the data on the reduction of metal ions in the presence of polyphenols. It is seen that all compounds are capable of reducing copper (II) ions (Figure 3a,b). 

The most effective reducing agents are quercetin, taxifolin, eriodicyol, and catechin, whereas DfTf, hesperetin, and naringenin produce a weaker copper-reducing effect. Thus, except for DfTf, the compounds containing a catechol fragment in the structure exhibit high copper-reducing activity.

These four compounds (quercetin, taxifolin, eriodicyol, and catechin) also effectively reduce iron ions (Figure 3c,d). It was found that catechin in the concentration range of 20–30 µM exhibits a higher iron-reducing activity than the other compounds (Figure 3c,d). As an example, Figure 3c presents the time-dependent reduction of iron (III) ions in the presence of polyphenols at a concentration of 20 μM. It is seen that eriodictyol exhibits a higher iron-reducing activity than taxifolin. There is no significant difference in the iron-reducing activity between eriodictyol and quercetin and between quercetin and taxifolin. The concentration of reduced iron ions in the presence of hesperetin and naringenin is low. DfTf is capable of reducing iron (III) ions; however, the concentration of iron (II) ions is significantly lower than in the presence of catechol-containing flavonoids and depends little on the concentration of this polyphenol in the system, which has been found earlier by us at pH 5.4 [31]. It can be suggested that this is related to the iron-binding capacity of DfTf. To verify this assumption, we estimated the rigidity of the complex formed between DfTf and Fe (II).

### 3.3. Iron-Binding Properties of DfTf

**Figure 4:**
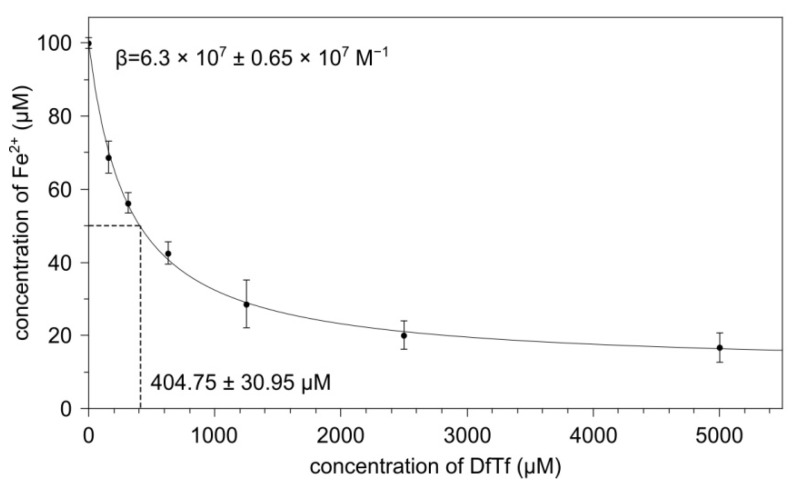
Effect of a conjugate of taxifolin with glyoxylic acid (DfTf) on the formation of the ferrozine-iron (II) complex.

We have found earlier that DfTf is capable of binding iron (II) ions [31]. Here, we studied the ability of DfTf to bind iron (II) ions in the presence of ferrozine, a strong chelator of the corresponding ions. It was shown that the addition of DfTf to an iron (II) sulfate solution leads to the appearance of a new absorption band (λ_max_ 460 nm) not characteristic for this polyphenol, indicating the formation of the polyphenol-Fe (II) complex. The addition of ferrozine to the system after 30 min of incubation leads to the binding of free iron and iron weakly bound with polyphenols and the formation of a ferrozine–Fe (II) complex with the absorption maximum at 562 nm. Changes in the optical density at this wavelength at different molar ratios of DfTf/ferrozine indicate that these compounds compete with each other for the binding of iron (II) ions (Figure 4). Based on these data, the apparent binding constant of the DfTf-Fe (II) complex was estimated to be 6.3 × 10^7^ M^−1^. 

### 3.4. Lipid Peroxidation Inhibitory Activity

**Figure 5:**
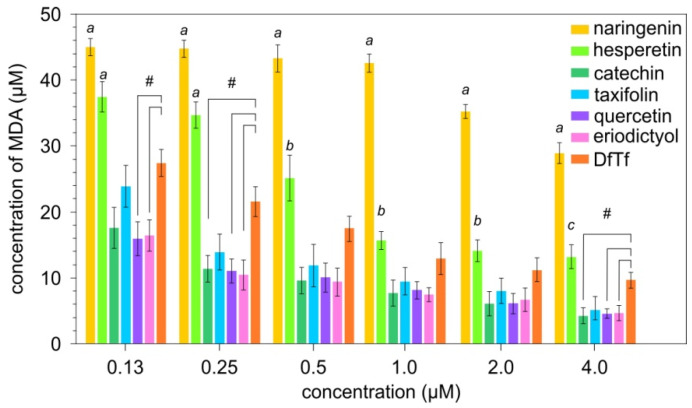
Effect of polyphenols on lipid peroxidation. ^a^ differences are significant compared with other compounds; ^b^ differences are significant compared with catechin, quercetin, eriodictyol, and naringenin; ^c^ differences are significant compared with other compounds, with the exception of DfTf; ^#^ significant differences between the compounds, *p* < 0.05.

It was found that quercetin, taxifolin, eriodictyol, and catechin are the most effective LPO inhibitors, which have similar properties in the concentration range used (Figure 5). DfTf in the range of lower concentrations inhibits LPO to a lesser extent than quercetin, catechin, and eriodicyol. However, there is no significant difference between the inhibitory effects of DfTf and taxifolin. In turn, DfTf at low concentrations more strongly inhibits LPO than hesperetin. Hesperetin inhibits LPO in a significantly weaker manner than all flavonoids containing a catechol fragment in their structure. Naringenin produces the least inhibitory effect (Figure 5).

## 4. Discussion

The results of the study indicate that compounds containing hydroxyl groups in the B ring and being in the *ortho*-position relative to each other (the catechol fragment) have the most effective antioxidant activity (DfTf, eriodictyol, quercetin, taxifolin, catechin). The methylation of the hydroxyl group at the 4′-position (hesperetin) or the absence of the hydroxyl group at the 3′-position of the B ring (naringenin) leads to a significant reduction in activity. The presence of the 2,3-double bond, or the carbonyl group at the C4 atom, or the aliphatic hydroxyl group at the 3-position of the C ring affects the antioxidant properties of these compounds substantially less. These results agree well with the literature data indicating that quercetin exhibits high scavenging activity toward radicals generated in the aqueous phase (ABTS and DPPH radicals). Catechin and taxifolin show a lower radical scavenging activity than quercetin, whereas hesperetin and naringenin possess a lower antiradical activity than flavonoids mentioned above [35,36]. In spite of the fact that the order of efficacy of flavonoids as radical scavengers can change depending on experimental conditions and methods used by the authors, the most important structural feature of high antiradical activity for these compounds remains the same: the presence of the *ortho*-dihydroxy group in the B-ring [35,36]. DfTf is a dimer consisting of two taxifolin units, which are linked via the carboxymethine bridge at the C-6 and C-8 positions of the A ring [31]. Consequently, DfTf contains twice as many hydroxyl groups in the structure as the parent flavonoid and, as indicated above, shows the highest hydrogen peroxide scavenging activity. Based on these data and on our previous results, it can be concluded that the effectiveness of DfTf against ROS present in the aqueous phase is higher than that of the parent flavonoid taxifolin [31] and its structural analogs (quercetin, eriodictyol, catechin, hesperetin, and naringenin).

Although the properties of the products of condensation and polymerization of flavonoids remain poorly studied [37,38], there is evidence that these compounds exhibit higher antioxidant, antimicrobal, and enzyme inhibitory activities than parent flavonoids [38,39,40,41,42,43,44]. In particular, the polycondensates of catechin-aldehyde and poly(rutin) show higher superoxide-anion scavenging activity and inhibit the oxidation of human low-density lipoprotein to a greater extent than the monomeric form of flavonoids [39,41,42]. Poly(naringenin) has a greater activity in reducing ABTS and DPPH radicals than naringenin [44]. Proanthocyanidins found in various plants exhibit potent antioxidant activity, which positively correlates with the degree of their polymerization [45,46,47,48]. The products of reactions between quercetin and methylglyoxal (mono- and di-MGO quercetin adducts) retain the ability to scavenge DPPH radical and carbonyl compounds [19]. Thus, the condensation of flavonoids with toxic lipid peroxidation products leads not only to the utilization of the latter, but also to the formation of compounds exhibiting high antioxidant activity.

The antioxidant activity of polyphenols in turn determines their capacity to reduce transition metal ions, which can participate in the Fenton reaction in the presence of hydrogen peroxide, resulting in the initiation and branching of radical chains [49,50]. It is worth noting that, in systems containing polyphenol and transition metal ions, such as Fe (III) and Cu (II), several processes affecting each other may take place simultaneously. In particular, it is known that polyphenols bind transition metal ions, forming complexes with different stoichiometry, a process which highly depends on experimental conditions [32,51,52,53,54]. The redox process leads to changes in the composition and the ratio of the components present in the system. As a result, various complexes are composed, among other things, of oxidized forms of flavonoids and reduced metal ions form [54,55]. On the other hand, complex formation leads to changes in the redox potential of the system and a shift of the equilibrium of the redox reaction. Reagents used for the estimation of the metal-reducing activity of compounds, such as ferrozine and BCDS, firmly bind reduced metal ions (the constant of binding of ferrozine to Fe^2+^ is logβ = 15.5 [56] and that of BCDS to Cu^+^ is logβ = 19.8 [57]), minimizing the effects of processes proceeding simultaneously on the result of the measurement. Nevertheless, if a polyphenol is capable of firmly binding metal ions, it may compete with the reagent (ferrozine, BCDS) for the binding of the corresponding metal ions [53,55,58,59,60,61,62]. In these cases, the concentration of ions that was reduced in the presence of the polyphenol may also be considered as a measure of its redox activity in the reaction mixture and can be used to compare the metal-reducing activity of this polyphenol and other compounds. In turn, the model system containing reagents competing with polyphenols for metal ions offers an additional tool for studying polyphenol–metal ion interaction. The data being obtained by means of these systems form the basis necessary for a better understanding of the mechanisms underlying the pro- and antioxidant effects of polyphenols as well as a switch from antioxidant to prooxidant properties of these compounds [49,50]. 

An examination of the metal-reducing activity of polyphenols showed that copper (II) and iron (III) ions are reduced most effectively in the presence of four compounds containing a catechol fragment: catechin, quercetin, eriodictyol, and taxifolin. In the literature, there is evidence indicating a high metal-reducing activity of catechol-containing compounds (quercetin, taxifolin, catechin) [32,63,64]. However, the order of metal-reducing efficacy of flavonoids may change depending on pH, the flavonoid/metal ion molar ratio, and the time interval [63,64,65]. Most likely, in the first two cases, the changes in the redox potentials of the systems play a key role, while in the last case the decisive factor is the kinetics of the redox reactions [64]. In general, the results obtained here and the available literature data demonstrate high metal-reducing activity of polyphenols containing the catechol group [32,63,64]. DfTf contains two catechol moieties in the structure. Nevertheless, the metal-reducing activity of this compound is lower than that of catechol-containing flavonoids, suggesting that this polyphenol is less likely to act as a prooxidant. The metal-reducing capacity of poly(catechin)s condensed through acetaldehyde was also lower than that of the parent flavonoid and decreased as their molecular weight increased [39]. The copper-reducing activity of poly(naringenin) was lower than that of naringenin [44]. On the other hand, the metal-reducing power of proanthocyanidins increased with increasing size of polyphenols [58]. 

On the whole, our results support the data indicating that polyphenols (including DfTf) exhibit higher reducing capacity toward copper ions than toward iron ions [32]. It is significant that the concentration of iron (II) ions in the presence of DfTf depends only insignificantly on its concentration in the system, which we have shown earlier at pH 5.4 [31]. This effect is not revealed in the presence of other catechol-containing compounds studied (taxifolin, eriodictyol, catechin, and quercetin) and may be associated with the metal-binding capacity of DfTf and its oxidized forms. This assumption is supported by the fact that ferrozine and DfTf compete with each other for the binding of Fe (II) ions; in this case, DfTf rather firmly binds these ions. To date there are no studies devoted to the evaluation of the stability of complexes formed between the conjugates of flavonoids with carbonyl compounds and transition metal ions. However, in the literature there is evidence indicating that some polyphenols compete for binding of the corresponding ions with well-known chelators, including ferrozine. These are baicalein (acidic and neutral pH) [53,59], quercetin (neutral pH) [53,55,60], isorhamnetin and tamarixetin (neutral pH) [61], isoquercetin (slightly acidic and neutral pH) [60], proanthocyanidins [58], and tannic acid [62]. Therefore, both monomeric and polymeric polyphenols are capable of firmly binding transition metal ions. Nevertheless, the data available in the literature do not enable one to directly estimate the impact of condensation reactions on the metal-binding capacity of the polyphenolic fraction. Here, we demonstrate for the first time that the interaction of taxifolin with glyoxylic acid leads to the formation of a product (DfTf) exhibiting higher metal-binding activity in comparison not only to the parent flavonoid but also to its structural analog quercetin, which possesses higher metal-binding capacity than taxifolin [53]. Taking into account the structural features of polyphenols and the data presented in the literature, several iron binding sites of DfTf can be proposed: (1) the 4-carbonyl group in the C ring and the 5-hydroxyl group of the A ring; (2) the 4-carbonyl group in the C ring and the 3-hydroxyl group of the C ring; and (3) the 3′- and 4′-hydroxy groups of the B ring [49,51,53,66]. Our previous results have shown that naringenin lacking the 3-hydroxyl group in the C ring and 3′-hydroxy groups in the B ring [51,52] are able to form complexes with iron (II) and copper (I) ions, suggesting that the coordination of metal ions involves the carbonyl and 3-hydroxyl groups. It may be assumed that the coordination of metal ions in DfTf-iron complexes occurs at the same positions. Taxifolin also forms complexes with iron (II) and copper (I) ions [51,52]. With due regard for the stoichiometry of the complexes, it may be concluded that, in contrast to naringenin, one molecule of taxifolin is capable of binding two metal ions [51,52], indicating that taxifolin has two coordination sites. Presumably, DfTf has similar coordination sites. In favor of this suggestion are our previous data indicating that DfTf is able to bind up to four iron (II) ions [31]. Supposing that one metal ion is coordinated at the carbonyl group in the fourth position of the C ring and at the 5-OH-group, it is more likely that the other ion is coordinated at the 3′- and 4′-hydroxy groups of the B ring. Nevertheless, the involvement of the 3-hydroxyl and 4-oxo groups in the complex formation by the polyphenols cannot be excluded.

A study of LPO in the presence of polyphenols and iron (II) ions showed that quercetin, catechin, eriodictyol, and taxifolin (compounds containing a catechol fragment) are effective inhibitors of LPO and exert similar inhibitory effects. Other studies on the effects of flavonoids on the lipid peroxidation demonstrated a different order of the effectiveness of these compounds, which depends on the system used [67,68]. Thus, Shahidi and coworkers have shown that quercetin and taxifolin exhibit similar antioxidant activity in a lipid system [69], while in another study quercetin inhibited the lipid peroxidation to a lesser extent than taxifolin [70]. Quercetin better protects lipids from oxidation induced by ultraviolet radiation or Fe (II) than catechin [68]. In addition, quercetin prevents the autooxidation of rat cerebral membranes better than hesperetin and naringenin [67]. On the other hand, hesperetin prevents linoleate peroxidation induced by Fe (II) better than quercetin [67]. On the whole, the data indicate that compounds containing the *ortho*-dihydroxy group exhibit a high lipid peroxidation inhibitory activity [36,71,72]. 

Thus, our results indicate that catechol-containing flavonoids exhibit high antioxidant activity against ROS present in the aqueous phase and ROS generated in the biphasic system. In contrast, DfTf, which shows the highest antioxidant activity in the aqueous phase, inhibits LPO worse than quercetin, eriodictyol, and catechin. Presumably, this is related to the physicochemical properties of these polyphenols (Table 2). As seen from the table, DfTf is most hydrophilic among all compounds examined. It can be suggested that the more hydrophobic quercetin, eriodictyol, and catechin accumulate in the lipid to a greater extent than hydrophilic DfTf.

**Figure 6:**
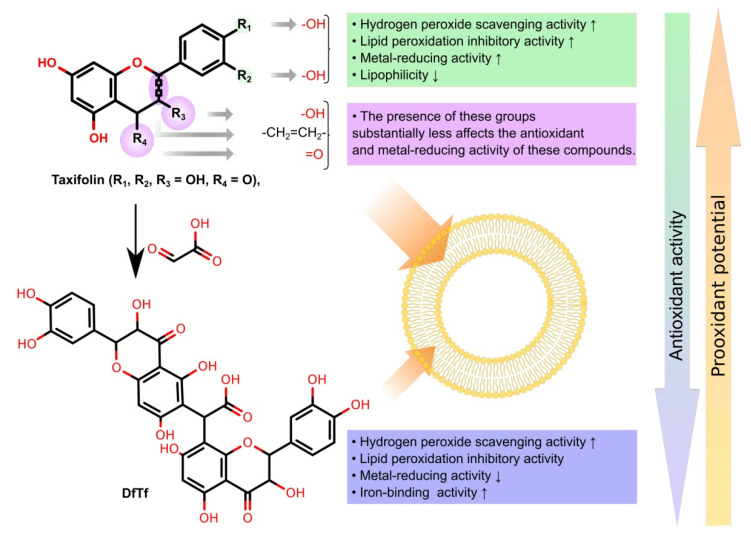
Summary of the structure–activity relationships for the antioxidant activities of the polyphenols being tested.

This enhances the antioxidant effect produced by these compounds, namely, the inhibition of LPO. At the same time, no significant difference was found between the inhibitory effects of DfTf and taxifolin. In addition, DfTf inhibits LPO to a greater extent than hesperetin. In general, the results obtained indicate that DfTf may act as a relatively strong antioxidant in biphasic systems. As mentioned above, poly(catechin)s and poly(rutin) inhibit the oxidation of human low-density lipoprotein [39,42]. Additionally, these polymers protect endothelial cells from oxidative injury induced by AAPH AAPH [39,42]. Water-soluble oligomers can also exert high antioxidant activity in oil-in-water emulsion systems [58,83]. It has been suggested that, owing to their amphipathic structure, hydrophilic polyphenols can interact with lipids, which can lead to the localization of these compounds at the lipid interface where they can efficiently inhibit LPO [58,83]. Probably, the location of DfTf to some extent influences its LPO inhibitory activity. Meanwhile, the metal-binding properties of the product may play a key role in the protection of lipids from oxidation. A summary of the structure–activity relationships for the antioxidant activities of the polyphenols being tested is shown in Figure 6.

## 5. Conclusions

The results obtained indicate that quercetin, taxifolin, eriodicyol, and catechin, compounds containing a catechol group, exhibit a higher antioxidant activity than hesperetin and naringenin. The product of the condensation of taxifolin with glyoxylic acid contains two catechol fragments in its structure. However, the relative efficiency of this compound differs in various systems. The product exhibits the highest scavenging activity toward hydrogen peroxide, a lower metal-reducing activity and a higher iron-binding activity than catechol-containing flavonoids (the product competes with ferrozine for binding to iron ions); its lipid peroxidation inhibitory activity is comparable with that of taxifolin. Our previous results have shown that the product is more hydrophilic than taxifolin, indicating different distribution of these compounds in biphasic systems. As a whole, these data indicate that the condensation of flavonoids with toxic carbonyl compounds leads not only to the utilization of the latter but also to the formation of products that exhibit high antioxidant activity and, probably, are capable of scavenging additional molecules of carbonyl compounds. In turn, the properties of parent flavonoids and products as well as the conditions under which they show the maximum antioxidant activity can vary. Thus, in the course of condensation reactions, the composition and the antioxidant profile of the polyphenols fraction change. In addition, the bioavailability of polyphenols and carbonyl compounds involved in these reactions as well as that of food components such as minerals (owing to the metal-binding properties of products) can change.

## Figures and Tables

**Table 1:** Polyphenol concentrations necessary for the 50% inhibition of chemiluminescence (IC_50_).

Polyphenols	IC_50_, µM
DfTf	0.48 ± 0.04 *
eriodictyol	0.73 ± 0.05
quercetin	0.75 ± 0.05
taxifolin	0.76 ± 0.05
catechin	0.93 ± 0.09
hesperetin	2.17 ± 0.15 *
naringenin	34.2 ± 4.32 *

The values are the means ± standard deviation of ten independent experiments. * Significantly different from other compounds, *p* < 0.05.

**Table 2:** Physicochemical parameters of polyphenols being tested.

Polyphenols	Solubility, mg/mL	logP	logD_7.4_
DfTf	more than 12 mg/mL	1.13 ± 0.12 [31]	-
taxifolin	0.87 [73] 1.0 [74]	1.57 ± 0.03 [52]	0.96 ± 0.02 [52]
quercetin	0.060 [75]	1.82 ± 0.32 [76] 1.26 ^1^ [72]	0.76 ± 0.09 [77]
catechin	4.54 [78]	1.31 ^1^ [72]	-
hesperetin	0.001 [79]	2.6 [80]	-
naringenin	0.00438 [81]	2.30 ± 0.18 [52] 2.60 ± 0.03 [76] 1.60 ^1^ [72] 2.52 [80]	2.18 ± 0.06 [52] 2.09 ± 0.14 [82]
eriodictyol	0.070 [75]	2.27 ± 0.02 [76] 2.02 [80]	-

^1^ Experiments were carried out at 50 °C.

## Data Availability

Data is available within the article and supplementary materials.

## Supplementary Materials

The following are available online at https://www.mdpi.com/article/10.3390/antiox10081262/s1, Materials and Methods S1: the calculation of the apparent binding constant.
