# Supplementary material for: Comparison of Antioxidant Properties of a Conjugate of Taxifolin with Glyoxylic Acid and Selected Flavonoids
Source: Antioxidants (Basel). 2021 Aug 8;10(8):1262. doi: 10.3390/antiox10081262 (PMC8389318; doi:10.3390/antiox10081262)
Supplement: Supplementary file 1 [file antioxidants-10-01262-s001.zip › antioxidants-1317845-supplementary.pdf]

Supporting Information for Comparison of Antioxidant Properties of a Conjugate of Taxifolin with Glyoxylic Acid and Selected Flavonoids

V. S. Shubina, V. I. Kozina and Yu. V. Shatalin

Institute of Theoretical and Experimental Biophysics, Russian Academy of Sciences, Institutskaya 3, 142290 Pushchino, Russia

For a mixture containing two compounds that compete for binding to iron(II) ions, the following equilibria are established:

(1) Fe(II) + 3 FZ = Fe(II)·FZ_3; K_b = [Fe(II)·FZ_3]$_e$/[Fe(II)]$_e$ × [FZ]$_e^3$;

(2) Fe(II) + DfTf = Fe(II)·DfTf; K_c = [Fe(II)·DfTf]$_e$/[[Fe(II)]$_e$ × [DfTf]$_e$;

where FZ is ferrozine; DfTf is a conjugate of taxifolin with glyoxylic acid; K_b and K_c are the binding constant of Fe(II) and ferrozin and Fe(II) and DfTf, respectively; and [...]$_e$ is the equilibrium concentration of each species.

The ratio of the binding constants is expressed as:

K_b/K_c = ([Fe(II)·FZ_3]$_e$/[Fe(II)·DfTf]$_e$) × ([DfTf]$_e$/[FZ]$_e^3$) =>

K_c = K_b × ([FZ]$_e^3$/[DfTf]$_e$) × ([Fe(II)·DfTf]$_e$/[Fe(II)·FZ_3]$_e$).

where $K_b = 3.65 \times 10^{15}$ M^{-2} [56]

In the case that equal amounts of [Fe(II)·DfTf] and [Fe(II)·FZ_3] complexes are formed, we obtain:

[Fe(II)·FZ_3]$_e$ ≈ [Fe(II)·DfTf]$_e$ ≈ 50×10^{-6} M; [DfTf]$_0$ = 404.75×10^{-6} M (the initial concentration of DfTf was found by interpolating the graph on Fig. 4);

Thus, [DfTf]$_e$ ≈ [DfTf]$_0$ - [Fe(II)·DfTf]$_e$ = 404.75×10^{-6} M – 50×10^{-6} M = 354.75×10^{-6} M.

Taking into account that the initial concentration of ferrozine ([FZ]$_0$) was 333×10^{-6} M, we have:

[FZ]$_e$ ≈ [FZ]$_0$ – 3 × [Fe(II)·FZ_3]$_e$ = 333×10^{-6} M – 3 × 50×10^{-6} M = 183×10^{-6} M.

Thus, $K_c = 6.3 \times 10^7$ M^{-1}.
